# Open Globe: Corneal Laceration Injury with Negative Seidel Sign

**DOI:** 10.5811/cpcem.2018.4.38086

**Published:** 2018-06-12

**Authors:** Kyle Couperus, Andrew Zabel, Morohunranti O. Oguntoye

**Affiliations:** * Madigan Army Medical Center, Department of Emergency Medicine, Tacoma, Washington; † William Beaumont Army Medical Center, El Paso, Texas; ‡ Kimbrough Ambulatory Care Center, Department of Ophthalmology, Fort Meade, Maryland

## CASE PRESENTATION

**Image 1:**
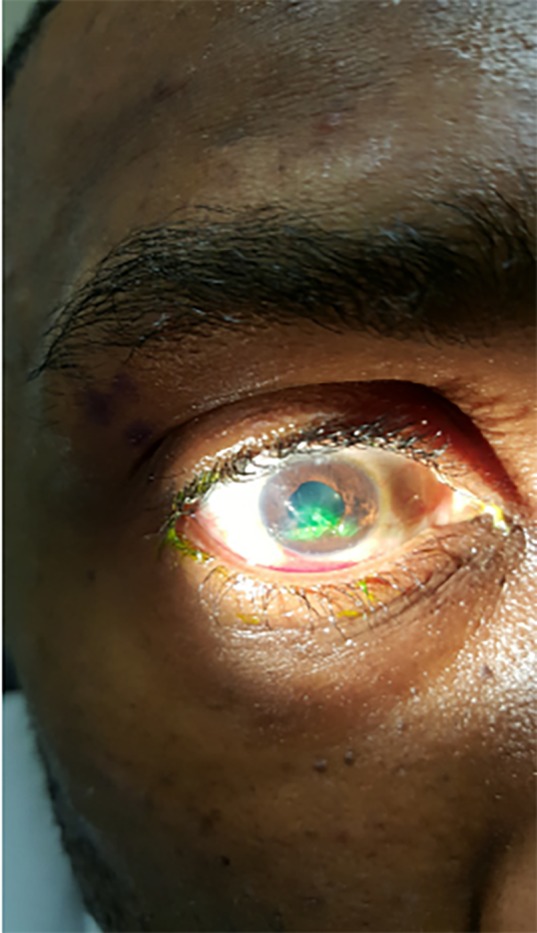
Stellate corneal laceration with negative Seidel Sign.

**Image 2:**
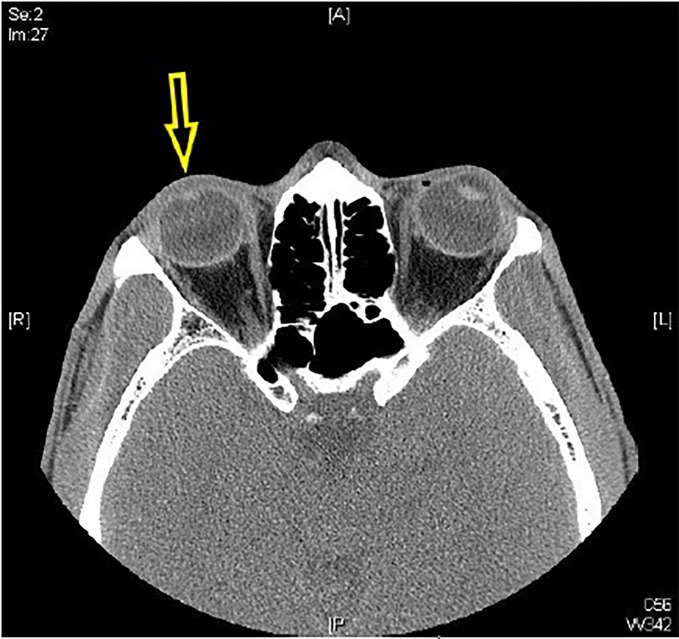
Computed tomography of the orbits, axial view, revealing right flat anterior chamber (yellow arrow) with iris abutting the cornea.

We present a 31-year-old male who sustained an isolated stellate corneal laceration associated with an open globe injury. The patient presented with mild, right eye pain one hour after glass was sustained to the face during a motor vehicle collision. Visual acuity was 20/100 (baseline 20/20), but no obvious facial or ocular trauma was noted. Extraocular movements were intact. Slit lamp examination revealed a central stellate corneal laceration, peaked 4mm non-reactive pupil, flat anterior chamber, and a falsely negative Seidel sign (Image 1). Intraocular pressure was not measured given the nature of the injury. Computed tomography (CT) orbits revealed a flat anterior chamber (Image 2). The patient was placed in an eye shield, treated for nausea/pain, initiated on antibiotics with levofloxacin, and updated on tetanus; ophthalmology then completed a surgical repair.

## DISCUSSION

Ocular trauma accounts for roughly 3% of emergency department visits and is a major cause of unilateral visual impairment and permanent visual loss in young individuals.^1^,^2^ Open globe injuries occur more commonly in males and should be in the differential diagnosis with any injury involving high-velocity metal or glass.^1^,^3^ Penetrating mechanisms tend to be more common in the young, while a blunt mechanism is more common in the elderly.^1^,^3^ Exam findings can be subtle. Classic teaching revolves around Seidel’s sign; it is not sensitive, but it is specific.^4^ A globe rupture with false negative Seidel sign is a rare but known occurrence when ocular contents “plug” the opening, as seen in this patient, preventing aqueous outflow and causing a falsely negative Seidel sign. Other suggestive exam findings include a peaked pupil, poorly reactive pupil, flat anterior chamber, and visual acuity changes.^4^ Despite poor sensitivity, CT is very specific and can be helpful when identifying open globe injuries.

Documented patient informed consent and/or Institutional Review Board approval has been obtained and filed for publication of this case report.

**CPC-EM Capsule**

What do we already know about this clinical entity?

Open globe rupture is an ophthalmologic emergency. Speedy recognition by the emergency department provider and ophthalmologic intervention are essential to restoring functional outcome.

What is the major impact of the image(s)?

A globe rupture with false negative Seidel sign is a rare but known occurrence when ocular contents “plug” the opening, as seen in this patient image.

How might this improve emergency medicine practice?

The case highlights the importance of good clinical exam after ocular trauma. Providers should consider further workup with computed tomography imaging with any injury involving high-velocity metal or glass.
